# Supplementary material for: MetaRibo-Seq measures translation in microbiomes
Source: Nat Commun. 2020 Jun 29;11:3268. doi: 10.1038/s41467-020-17081-z (PMC7324362; doi:10.1038/s41467-020-17081-z)
Supplement: Supplementary file 10 — Supplementary Data 7 [file 41467_2020_17081_MOESM10_ESM.zip › File2/Confidence_High_Taxonomy/31213_out.krona.html]

Javascript must be enabled to view this page.

members
magnitude
magnitudeUnassigned
count
unassigned
taxon
rank

31213\_out

3

2
superkingdom
3

phylum
3

SRS012849\_contig\_number\_contig-100\_1950.1950
1
1239

class
2
526524

2
order
526525

128827
family
2

genus
2
135858

2

SRS015431\_contig\_number\_10662SRS019068\_contig\_number\_113915
species
1946309
